# First report of the infection of insecticide-resistant malaria vector mosquitoes with an entomopathogenic fungus under field conditions

**DOI:** 10.1186/1475-2875-10-24

**Published:** 2011-02-02

**Authors:** Annabel FV Howard, Raphael N'Guessan, Constantianus JM Koenraadt, Alex Asidi, Marit Farenhorst, Martin Akogbéto, Bart GJ Knols, Willem Takken

**Affiliations:** 1 Laboratory of Entomology, Wageningen University and Research Centre, P.O. Box 8031, 6700 EH Wageningen, The Netherlands; 2 London School of Hygiene and Tropical Medicine, Keppel Street, WC1E 7HT, London, UK; 3 Centre de Recherche Entomologiques de Cotonou (CREC), 06 BP 2604 Cotonou, Benin; 4 Div. Infectious Diseases, Tropical Medicine & AIDS, Academic Medical Center, F4-217 Meibergdreef 9, 1105 AZ Amsterdam, The Netherlands

## Abstract

**Background:**

Insecticide-resistant mosquitoes are compromising the ability of current mosquito control tools to control malaria vectors. A proposed new approach for mosquito control is to use entomopathogenic fungi. These fungi have been shown to be lethal to both insecticide-susceptible and insecticide-resistant mosquitoes under laboratory conditions. The goal of this study was to see whether entomopathogenic fungi could be used to infect insecticide-resistant malaria vectors under field conditions, and to see whether the virulence and viability of the fungal conidia decreased after exposure to ambient African field conditions.

**Methods:**

This study used the fungus *Beauveria bassiana *to infect the insecticide-resistant malaria vector *Anopheles gambiae s.s *(Diptera: Culicidae) VKPER laboratory colony strain. Fungal conidia were applied to polyester netting and kept under West African field conditions for varying periods of time. The virulence of the fungal-treated netting was tested 1, 3 and 5 days after net application by exposing *An. gambiae s.s. *VKPER mosquitoes in WHO cone bioassays carried out under field conditions. In addition, the viability of *B. bassiana *conidia was measured after up to 20 days exposure to field conditions.

**Results:**

The results show that *B. bassiana *infection caused significantly increased mortality with the daily risk of dying being increased by 2.5× for the fungus-exposed mosquitoes compared to the control mosquitoes. However, the virulence of the *B. bassiana *conidia decreased with increasing time spent exposed to the field conditions, the older the treatment on the net, the lower the fungus-induced mortality rate. This is likely to be due to the climate because laboratory trials found no such decline within the same trial time period. Conidial viability also decreased with increasing exposure to the net and natural abiotic environmental conditions. After 20 days field exposure the conidial viability was 30%, but the viability of control conidia not exposed to the net or field conditions was 79%.

**Conclusions:**

This work shows promise for the use of *B. bassiana *fungal conidia against insecticide-resistant mosquitoes in the field, but further work is required to examine the role of environmental conditions on fungal virulence and viability with a view to eventually making the fungal conidia delivery system more able to withstand the ambient African climate.

## Background

Although the distribution of efficient malaria control tools, such as long lasting insecticide-treated bed nets (ITN) and effective chemotherapy, appear to be successfully reducing the number of people killed by malaria [1], problems still remain. As with many previously-effective anti-malarial drugs, resistance to artemisinin derivatives has emerged on the Thai-Cambodian border [2] and insecticide resistance in mosquitoes is widespread. Insecticide resistance refers to the ability of an insect to tolerate doses of an insecticide that would prove lethal to the majority of individuals in a normal population of the same species. Inheritable resistance traits develop by selective pressure exerted on a mosquito population. Fast-acting insecticides exert strong selection pressures, and the short generation time and prolific progeny characteristic of the mosquito lifecycle is well suited for quick development of resistance. Over 50 species of *Anopheles *are reported to be resistant to insecticides [3].

Several different strategies have been proposed to tackle insecticide resistance. Currently the World Health Organization (WHO) recommends the simultaneous use of different control tools and this forms the basis of integrated vector management (IVM) [4]. In addition, the rotation of different insecticides has been tested [5] and the use of novel insecticides alone [6,7] or in a mosaic with existing insecticides [8] has also been proposed. Solutions involving the entomopathogenic fungi *Metarhizium anisopliae *and *Beauveria bassiana *have also been put forward [9], and a combination of permethrin and entomopathogenic fungi showed a synergistic effect on malaria vector mortality [10].

The use of entomopathogenic fungi alone has also shown promise in terms of insecticide resistance management. Insecticide-resistant *Anopheles gambiae *(Diptera: Culicidae) mosquitoes were significantly more susceptible to fungal infection than the insecticide-susceptible strain [11]. The tendency to kill insecticide-resistant mosquitoes faster than insecticide-susceptible ones should be of benefit when tackling insecticide resistance in the field, because fungal infection will quickly remove insecticide resistance genes from the population while leaving the insecticide-susceptible mosquitoes to breed, which is important for keeping the fungus "evolution-proof" [12] and could lead to insecticide resistance management without the need for further insecticide use. This is possible because entomopathogenic fungi kill mosquitoes at a slower rate than insecticides [12]. The benefits of this are essentially two-fold. Primarily, the slow speed of kill leads to a reduced selection pressure for resistance to the fungi [12] because the mosquitoes have some reproductive success before being killed [13]. In addition, because the malaria parasite takes >10 days to develop within the mosquito, even a relatively modest speed of kill can prevent malaria transmission as long as coverage (i.e. probability of fungal infection per feeding cycle) is high [12,14]. Pre-lethal effects of reduced feeding and fecundity [15] and impaired parasite development [16] will further impede malaria transmission.

Whilst the use of entomopathogenic fungi against mosquitoes has provided encouraging results under controlled laboratory conditions [11,16,17], and in the field [18-20], some issues need to be addressed. Because entomopathogenic fungi are themselves living organisms it is important to test whether they will survive and be effective under field conditions where the temperature and humidity fluctuate. This is especially true in light of recent studies that suggest the viability of entomopathogenic fungi is affected by temperature. A recent study showed that the viability of *M. anisopliae *sprayed onto glass slides and kept at 26°C had dropped from 100% viability on day zero to 10% by day 7 and 0% by day 21; the same study found that for *B. bassiana *the viability stayed above 85% even 70 days after being sprayed onto the glass slides [21]. When oil-formulated fungal conidia were applied to polyester netting and kept at 27°C, the viabilities of *M. anisopliae *and *B. bassiana *were significantly reduced both one day and again one week after application, although this may also have been due to the polyester netting substrate [11]. Lekimme *et al *[22] inoculated temperate and tropical strains of *B. bassiana *and *M. anisopliae *onto agar plates and held them at a range of temperatures to investigate thermotolerance. They found that at 35°C only one *M. anisopliae *strain and none of their *B. bassiana *strains grew [22]. Whilst field work has shown that entomopathogenic fungi can infect and kill malaria vectors [18,19], and reduce blood feeding behaviour in *Culex *mosquitoes [20], Scholte *et al *[18] found that fungal viability reduced from 96% to 63% after three weeks exposure to Tanzanian field conditions.

In a previous study, the suitability of polyester netting as a substrate onto which entomopathogenic fungi could be applied was tested with a view for field deployment. The results of that study showed that while the conidial viability was significantly reduced after exposure to controlled laboratory conditions, the effectiveness of the fungal treatment at killing mosquitoes (virulence) did not significantly deteriorate [11]. It is unknown why the viability was so affected without diminishing the virulence, but since virulence was not affected it was decided to move into field studies because virulence against mosquitoes is an important factor for potential vector control tools. In the present study, the virulence of *B. bassiana *towards an insecticide-resistant laboratory colony of the malaria vector *An. gambiae *was examined under field conditions. In addition, the effect of field exposure on conidial viability was determined. These experiments were undertaken in Benin, West Africa, because there is widespread insecticide-resistance among malaria vectors [23-25] that is rendering current control tools ineffective [26], and because new tools that could potentially tackle insecticide resistance need to be tested in the environment in which they are needed.

## Methods

### Mosquitoes

The mosquitoes used were *An. gambiae s.s. *VKPER. This is a pyrethroid-resistant strain that was initially collected from the Valley du Kou in Burkina Faso and then selected repeatedly to fix the *kdr *gene. This gene is linked to knockdown resistance to pyrethroids and DDT, and was first reported in West African mosquitoes in the early 1990s [27]. The VKPER strain has been maintained as a colony at the Centre de Recherche Entomologique de Cotonou (CREC) in Benin for 14 years and is subject to standard rearing. Approximately 400 larvae were kept in plastic bowls filled with two litres of distilled water and fed on locally purchased crushed dry cat food (100 mg of food per two litre bowl per day). Adult mosquitoes are fed on a honey-water mixture *ad libitum *and kept in standard sized mesh-covered cages in an insectary exposed to ambient climate conditions.

### Fungus

*Beauveria bassiana *(Balsamo) Vuillemin IMI 391510 was produced by initially growing the fungus in a liquid medium and then inoculating autoclaved barley flakes in mushroom spawn bags at Penn State University, USA. After being dried at ambient temperature and then stored in the refrigerator, dry *B. bassiana *conidia were suspended in the synthetic isoparaffinic hydrocarbon solvent ShellSol T ™ (Shell, The Netherlands). ShellSol T was selected because the delivery system of fungal conidia suspended in this solvent has been shown to be significantly more virulent to *An. gambiae s.s. *mosquitoes when compared to conidia suspended in other oils [28]. A Bürker-Türk haemocyte counter and light microscope (at ×400) were used to determine accurate conidial concentrations per ml ShellSol T. New suspensions were made for each experimental replicate.

### Net treatment with the fungal conidia and storage

The netting used was made of white 100% multifilament 150 denier warp-knitted polyester fibres with 12 holes per cm^2 ^(Vestergaard Frandsen, Switzerland). Netting was dipped into the *B. bassiana *conidia/ShellSol T suspensions and the treatment densities were estimated because the size of the net and the volume of fungal suspension that was absorbed into the net were known. This resulted in a treatment density of 4.6 × 10^12 ^viable conidia per m^2^. Control netting was treated with ShellSol T only.

In Cotonou, Benin, pieces of netting were treated with *B. bassiana *conidia as described above and kept under ambient field conditions out of direct sunlight in a well ventilated storage shed to the side of the laboratory. Indoor conditions were chosen as it is proposed that entomopathogenic fungi will be used to target host-seeking mosquitoes inside people's houses [18]. A temperature and humidity gauge was included to monitor the temperature and humidity ranges that the nettings were exposed to. The nets were treated and stored, and bioassays carried out, in June 2009 at the beginning of the wet season.

### Conidial viability in the field

Pieces of netting that had been held under field conditions in Cotonou, Benin for 2, 4, 7, 10, 13, 16 and 20 days were transported back to Wageningen University, The Netherlands, to score fungal viability. As a positive control, samples of the conidial suspensions that had been kept in a refrigerator were also transported back and tested. Forty-eight hours elapsed between removing the samples from the field conditions in Cotonou and putting them on agar plates at Wageningen University.

As a measure for conidial viability, the germination of spores on a rich agar medium was measured. Either a drop of the conidial suspension or 1 cm^2 ^of the treated netting was placed onto Sabouraud Dextrose Agar (SDA) plates. The SDA plates had 0.001% benomyl added so that accurate germination could be recorded; benomyl is a fungicide that restricts the hyphal growth without affecting germination [29]. These plates were then incubated at 27°C in the dark and germination was scored 24 hrs later using a light microscope at ×400. A conidium was scored as germinated if the germ tube was at least twice the length of the conidium. A minimum of 300 conidia were counted per plate.

### Cone bioassays with fungus-treated netting

**Figure 1:**
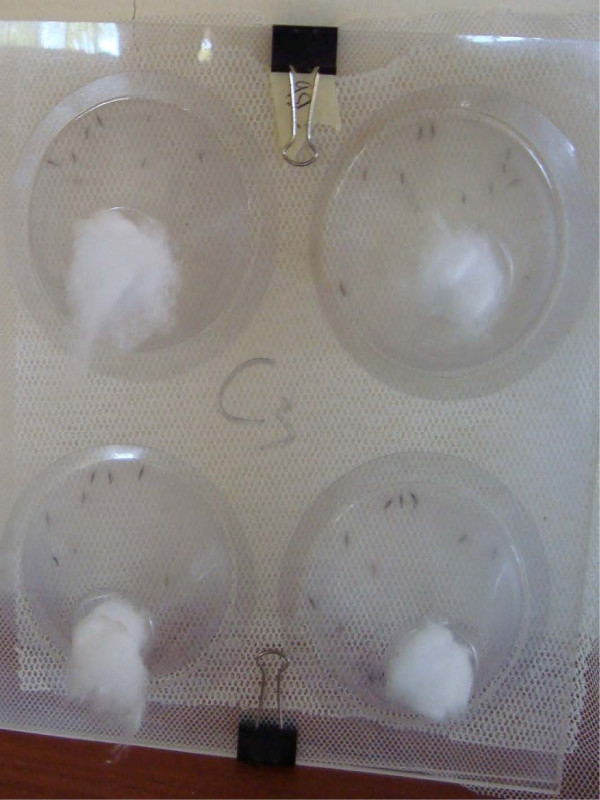
**Photograph of the WHO cone bioassay setup**. Photograph shows how the cones were suspended between two plastic sheets with holes in, with the mosquitoes directly contacting the netting. Fine mesh netting (best seen in the bottom of the photo) was used behind the treated net to prevent mosquitoes escaping.

To test fungal virulence after net treatment and storage, WHO cone bioassays were carried out in the field 1, 3 and 5 days post-net treatment. The cones and netting were set up so that mosquitoes had no alternative but to rest with their tarsi on the netting. This was achieved by suspending the treated pieces of netting between pieces of plastic with holes in them such that the plastic kept the cones in place but the holes ensured that the mosquitoes had to rest on the netting. Due to the possibility that mosquitoes may escape due to the relatively wide mesh of the fungus-treated netting, untreated finer mesh netting was placed behind the treated net (Figure 1).

Ten-to-twelve 2-3 day old non-blood fed *An. gambiae s.s. *VKPER females were introduced into each of the four replicate cones per treatment (control or *B. bassiana*). Because there was no previously published record of WHO cone bioassays being used to infect mosquitoes using entomopathogenic fungi applied to netting, it was estimated that an exposure time of 2 hours would allow the maximum chance of infection for the one day old fungal treatment, allowing any drop off in virulence on the nettings treated three or five days previously to be measured.

After the exposure period, mosquitoes were held in cups in the laboratory in Cotonou and given access to honey solution. Mortality was scored every 24 hours. For logistical reasons mosquito mortality could only be monitored up to day seven post exposure.

### Statistical analysis

To investigate whether the viability of fungal conidia significantly deteriorated with time simple linear regression analysis was carried out. Confidence intervals of the proportions were calculated using the normal approximation interval model. For the cone bioassays, the replicates were not significantly different from each other, so the data were pooled. For survival analysis, differences between the control and fungus-exposed mosquito survival rates were investigated using Cox' regression analysis. Mortality rates were given as hazard ratios (HR), which gives the average daily risk of dying relative to the control. All statistics were carried out in SPSS 17.0 [30] with α at 0.05.

## Results and Discussion

### Conidial viability in the field

**Figure 2:**
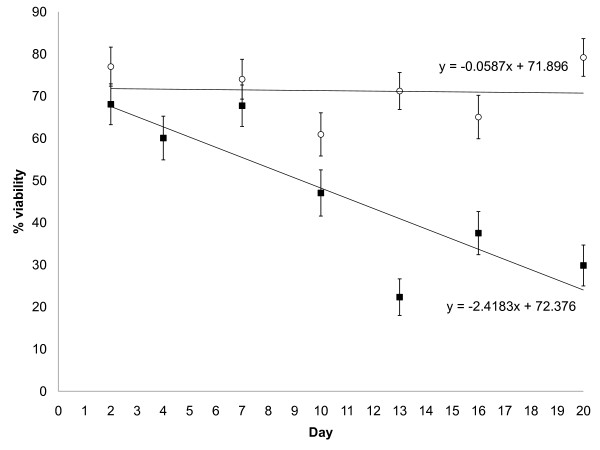
**Percentage viability of *Beauveria bassiana *conidia**. Conidia had either been applied to polyester netting and left in West African field conditions (closed squares) or were kept in the ShellSol T suspension in the refrigerator (open circles).

During the 20 day exposure of the treated pieces of netting to ambient field conditions in Benin, the temperature range in the storage area where the nets were held was 24.9 - 38.6°C with humidity ranging from 70 - >95%RH; daily means (±SE) were 30.0°C (±0.54) and 86%RH (±1.21). There was a steady and marked decrease in viability of the fungal conidia on the pieces of netting held under field conditions (Figure 2) (adjusted r^2 ^= 0.67, p = 0.015). Whilst the viability of the conidia on the netting decreased with exposure to the environmental conditions, the viability of the conidia remaining in suspension did not decrease (Figure 2) (adjusted r^2 ^= 0.2, p = 0.9). As a specific example, for the net held under field conditions for 20 days, the viability of the conidia on the netting was 30% but the viability of the conidia remaining in the ShellSol T solution used to treat that net was 79%. Thus either the polyester net or environmental conditions or a combination of the two was causing the conidial viability to decrease.

Fungal viability is a product of many variables including the production methods, the formulation used, the substrate treated and the climatic conditions. Whilst any of these variables can affect viability, these results showed that fungal viability decreased when treated pieces of polyester netting were left under ambient field conditions. Polyester netting has previously been shown to reduce conidial viability [11] and the reduction rate in the laboratory and field were similar. In addition, there is evidence that heat affects viability. A recent study found that *B. bassiana *conidia sprayed onto slides in the laboratory did not lose viability even after 70 days [21]. Darbro and Thomas [21] had kept the slides at 26°C because it was a "representative mean temperature for numerous malaria and dengue transmission areas" however, it is the thermo extremes that most affect biota and West Africa is hotter than many other malaria and dengue transmission areas. Therefore the difference between these findings and Darbro and Thomas' [21] findings relating to the viability of *B. bassiana *may be due to its lack of ability to withstand the thermo extremes (maximum temperature 38.6°C) encountered during the present study. Supporting evidence for this comes from results that indicate that 33°C is too hot for the sporulation, growth and viability of *B. bassiana *[31]. Further evidence can be found in a series of laboratory studies that reported relatively rapid losses in fungal viability after exposure to heat and humidity [11,22,32,33] and from *B. bassiana *gene-knockout studies that linked thermotolerance to viability [34,35]. In concurrence with these findings, Scholte *et al *[18] found that the viability of *M. anisopliae-*treated cotton sheets decreased from 96% in suspension to 63% three weeks after application in Tanzania, whereas the viability of the conidia remaining in suspension did not change [18], as was found in the present study.

The decline of *M. anisopliae *viability in the study in Tanzania [18] was less than was found for *B. bassiana *in the present study. Aside from the differences possibly arising from the different substrates (black cotton cloth vs. polyester netting), another possibility for the higher loss of conidial viability could be that the ShellSol T does not adequately protect the conidia to the same extent as the vegetable oil did in Tanzania [18]; in a contemporary field study it was found that ShellSol T evaporated releasing dry conidia one week after net treatment [20]. In addition, although the temperature ranges were fairly similar in this study and the study in Tanzania, lower humidity levels were experienced there [18]. Exposure to high humidity (as in the current study) or dry heat (as in Tanzania [18]) causes different conidial damage; dry heat causes DNA damage but humid-heat causes protein denaturation and membrane disorganization [32].

Another factor to consider when thinking about conidial viability is dose, and this study used a relatively high dose. If high enough doses are used then mosquitoes are still likely to contact enough viable conidia to contract a fatal infection even when fungal viability levels are low. This is because exposure time can be linked to virulence [17] because there appears to be a threshold number of viable conidia per unit surface area required for successful mosquito infection [36]. Of course, high doses may not be a cost-effective solution in many areas, and so high viability levels are still to be aimed at.

### Cone bioassays with fungus-treated netting

**Figure 3:**
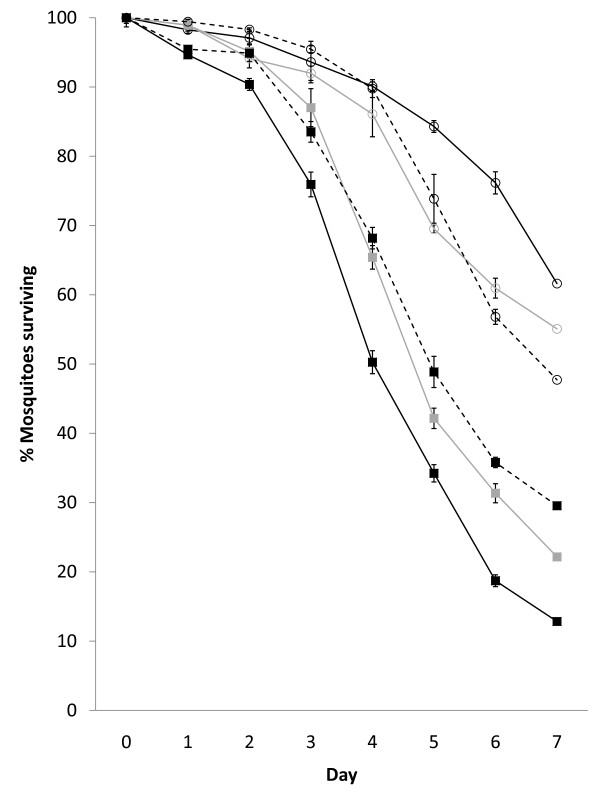
**Survival of *Anopheles gambiae s.s*. VKPER exposed in WHO cone bioassays to *B. bassiana*-treated netting**. Lines represent netting left in field conditions for 1 (solid black), 3 (solid grey) and 5 (dashed black) days before testing. Mean cumulative proportional survival (±SEM) for control-exposed mosquitoes (open circles) is compared to *B. bassiana*-exposed mosquitoes (solid squares).

The mean (±SE) temperature and humidity during the bioassay exposure periods were 29.2°C (±0.44) and 90.6%RH (±1.52), with ranges of 27.2-32.1°C and 78->95%RH respectively. Confirming previous results from the laboratory [10,11], *B. bassiana *was pathogenic to *An. gambiae s.s. *VKPER strain mosquitoes when exposed under field conditions (Figure 3). Significantly increased mortality for the *B. bassiana*-exposed mosquitoes (when compared to the control mosquitoes) was seen when the treatment on the nets was 1, 3 and 5 days old (Table 1). Despite being significantly different from the control for all the time points, the virulence of the *B. bassiana-*treated net held in field conditions significantly reduced with increased time in the field; mosquito mortality caused by the one day old fungal treatment on the net was significantly higher than the mortality caused by the 3-day old (HR = 1.33, p = 0.013) and 5-day old fungal treatments (HR = 1.49, p < 0.001). These results indicate a drop off of effectiveness with increasing time the fungal conidia spend exposed to ambient field conditions even over this relatively short trial period.

**Table 1:** Survival analysis of *Anopheles gambiae s.s. *VKPR mosquitoes exposed to the entomopathogenic fungus *Beauveria bassiana *in WHO cone bioassays

Age of fungal treatment on net (days)	Hazard Ratio (95% CI)	p-value
1	2.50 (1.94, 3.23)	<0.0001

3	2.24 (1.71, 2.93)	<0.0001

5	1.73 (1.32, 2.25)	<0.0001

Data show Cox regression Hazard Ratio outcomes (95% CI), p-values are relative to the relevant control

To the authors' knowledge, no previously published study has examined the use of entomopathogenic fungi against *Anopheles *adults using bioassays under field conditions and as such there was no field data with which to compare these findings. However, a list of previous laboratory studies that used *B. bassiana *to infect adult malaria vectors has recently been given [11]. Insecticide-susceptible malaria vectors infected using oil-formulated *B. bassiana *under laboratory conditions show significantly increased mortality [16,17,28,37]. In addition, a recent laboratory trial used the same insecticide-resistant mosquito strain infected with oil-formulated *B. bassiana *inoculated onto paper in tube bioassays [10]. This trial showed significantly increased mortality in the fungus-exposed laboratory *An. gambiae s.s. *VKPER strain, as well as in a wild-caught/F1 laboratory reared insecticide-resistant *An. gambiae *strain from Benin [10]. Another laboratory trial used the same insecticide-resistant mosquito strain and the same polyester netting as was used in the present field trial, but tube bioassays were used as opposed to the cones used in the field [11]. Nevertheless, a similar pattern can be seen, with significantly increased mortality in the *B. bassiana-*exposed mosquitoes when compared to the control mosquitoes [11]. The mortality of control-exposed mosquitoes was higher, and the fungal-induced mortality was slightly slower in the field trial, and this could be as a result of the different climatic conditions during the exposure periods. However, this slower speed of kill under field conditions can be of benefit because the theory of an evolution-proof malaria control tool is that slow acting substances are able to allow mosquitoes some reproductive success before they are killed [12,13].

What is of some concern is the apparent reduction of effectiveness with increasing time the fungal treatments spend under ambient field conditions. Because no decrease in virulence was found in a laboratory trial using the same mosquito strain/fungus/polyester net combination, but carried out over a longer trial period [11], it was concluded that the decrease in virulence over such a short trial period must be due to the ambient African climate. The reduction in virulence could also be linked to the decreasing viability of the *B. bassiana *conidia. Clearly this issue needs to be addressed in future work examining conidial delivery methods. In addition, a study with a 28-day trial period carried out in constant laboratory conditions found a reduction in virulence when *B. bassiana *conidia were applied to a variety of substrates [38]. Thus, entomopathogenic fungi conidial viability [11] and virulence [38] can be adversely affected even in constant laboratory conditions, and it would appear that West African climatic conditions add to this decline.

In spite of this reduction in virulence, the results from this study are encouraging because they demonstrate that insecticide-resistant malaria vectors can be infected with entomopathogenic fungi under ambient field conditions. In addition, this is the first study to use *B. bassiana *under field conditions to infect malaria vector mosquitoes. These results show that insecticide-resistant mosquitoes could be successfully controlled without the need for further insecticide use.

There is a growing body of evidence documenting encouraging results of the use of entomopathogenic fungi against mosquitoes under field conditions. A recent study found that wild insecticide-resistant *Culex *mosquitoes were not repelled by fungal treatments, and the blood feeding behaviour was significantly reduced in mosquitoes exposed to *B. bassiana-*treated window netting [20]. In addition, insecticide-susceptible malaria vector mosquitoes in Tanzania have been successfully infected with *M. anisopliae *leading to significantly shorter life spans of the infected mosquitoes [18,19]. More recently a natural *Lecanicillium muscarium *infection has been reported from a field-collected mosquito in Tanzania, and this fungus was pathogenic to *Aedes, Culex *and *Anopheles *mosquitoes under laboratory conditions [39]. More work on a larger scale needs to be carried out to ultimately monitor how the deployment of entomopathogenic fungi affects both mosquito population dynamics and malaria transmission.

Ultimately for mosquito control what is important is whether the fungi can still infect and kill mosquitoes in the field. The cone bioassay results show that these fungi were able to cause significant mortality to an insecticide-resistant strain of *An. gambiae *after the fungi had been held under field conditions. However, more work needs to be carried out before the operational use of these fungi can become a reality. Several major challenges remain before entomopathogenic fungi can be used for mosquito control. The encouraging findings from laboratory trials [9,11,15,16] need to be translated into field successes and effective and sustainable field delivery systems need to be developed. Future research on fungal production methods, possible micro-encapsulation, and testing new formulation and substrate combinations [38], should be carried out with a view to optimising these for eventual use in the field.

## Competing interests

The authors declare that they have no competing interests.

## Authors' contributions

AFVH designed and undertook the study, analysed the data and drafted the manuscript. RN'G aided in study design and supervised data collection. CJMK was involved in data analysis. AA and MF participated in data collection. MA supervised the field component of study. BGJK and WT coordinated the supervision of the study. All authors read and approved the final manuscript.

## Authors' Infomation

Raphael N'Guessan's email address is raphael.n'guessan@lshtm.ac.uk.
